# Registration accuracy of amyloid/tau-PET to brain MRI using modified SPM method

**DOI:** 10.1007/s12149-026-02159-3

**Published:** 2026-02-04

**Authors:** Yuma Iwao, Go Akamatsu, Muneyuki Sakata, Kei Wagatsuma, Kenji Ishii, Taiga Yamaya, Miwako Takahashi

**Affiliations:** 1 Institute for Quantum Medical Science, National Institutes for Quantum Science and Technology, 4-9-1, Anagawa, Inage-Ku, Chiba-Shi, Chiba, 263-8555 Japan; 2 Research Team for Neuroimaging, Tokyo Metropolitan Institute for Geriatrics and Gerontology, 35-2, Sakae-Cho, Itabashi-Ku, Tokyo, 173-0015 Japan; 3 Faculty of Health Sciences, Hokkaido University, North 12, West 5, Kita-Ku, Sapporo , Hokkaido 060-0812 Japan

**Keywords:** Registration, Amyloid tracers, Tau tracers, MRI, PET

## Abstract

**Objective:**

Mutual information-based algorithms, such as those in statistical parametric mapping (SPM), are widely used in multimodal image registration, but they may lose accuracy when tracer uptake patterns diverge from magnetic resonance imaging (MRI) contrast, as in amyloid and tau- positron emission tomography (PET). This study assessed the registration accuracy of MRI and amyloid/tau-PET images using SPM’s default settings and a modified approach (Modified SPM) incorporating a repeated optimization process. Registration accuracy was also examined in PET images without scatter/attenuation correction (uncorrected PET), mimicking PET-only systems lacking structural imaging for attenuation correction.

**Methods:**

Four PET tracers were analyzed: two amyloid tracers ([^11^C]PiB, [^1^⁸F]florbetapir) and two tau tracers ([^11^C]PBB3, [^1^⁸F]THK5351). The MR images were manually registered on the PET scans, then misaligned with known perturbations, and re-registered using the default SPM and the Modified SPM. The registration error was defined as the difference between the applied perturbations and re-registration parameters. A voxel size of 2.0 × 2.0 × 3.27 mm^3^ was used; errors less than one voxel were considered acceptable.

**Results:**

The average errors were less than one voxel overall; however, when stratified by PET-negative and PET-positive cases, rotational displacements were observed in tau-PET images with the default SPM only. The Modified SPM reduced these errors and improved the accuracy of uncorrected PET images.

**Conclusions:**

Registration accuracy between amyloid/tau-PET and MR images using default SPM was generally acceptable, although larger errors were observed in tau and uncorrected PET images. Incorporating a repeated optimization process (Modified SPM) improved registration robustness, particularly in these challenging cases.

**Supplementary Information:**

The online version contains supplementary material available at 10.1007/s12149-026-02159-3.

## Introduction

Anatomical localization in the central nervous system is crucial for evaluating alterations in neuronal function. Compared with positron emission tomography (PET), magnetic resonance imaging (MRI) provides more detailed anatomical information, such as tissue contrast between the white and gray brain matter. Therefore, combining PET and brain MRI facilitates accurate anatomical localization of PET images. Among the various registration methods, mutual information (MI)-based algorithms exhibit a robust performance [1, 2]. In a previous study, accuracy assessments demonstrated a registration error of 1.26 mm in the simulation experiments [3]. In a clinical [^18^F]fluorodeoxyglucose (FDG)-PET study, registration errors were found to be within 3 mm and 4° in 99% of images, even when substantial initial misalignments existed between MR and PET images [1].

The MI algorithm performs image registration by maximising the statistical dependence between images, even when acquired using different modalities. However, the registration accuracy may be compromised when the corresponding anatomical features are limited [2]. With the increasing clinical use of PET tracers targeting amyloid or tau deposition, notable differences in tracer distribution have been observed between negative and positive PET images. In typical amyloid-negative cases, the tracer intensity is higher in the white matter than in the gray matter. However, this distinction diminishes in positive cases as tracer accumulation in the gray matter becomes similar to that in the white matter. In tau-PET images, positive cases frequently exhibit tracer uptake confined to specific regions of the gray matter, further reducing corresponding features between MRI and tau-PET images and thereby diminishing the statistical dependence exploited by the MI algorithm. Therefore, evaluation of registration accuracy for these newly introduced amyloid and tau tracers is essential; however, investigations addressing this issue remain scarce, with only one study comparing the performance of registration software using [^18^F]florbetapir PET and MRI [2].

In this study, the registration accuracy between MR image and amyloid PET using [^11^C]PiB [4] and [^18^F]florbetapir [5, 6], as well as tau-PET using [^11^C]PBB3 [7] and [^18^F]THK5351 [8], was evaluated using the default settings of Statistical Parametric Mapping (SPM) [9]—a widely used software implementing MI-based algorithms. Furthermore, we addressed the tendency of Powell’s method, which is used in SPM, to converge to the local minima in multimodal image registration, where the algorithm may prematurely settle in a suboptimal position. To address this, we incorporated a repeated optimization process into the SPM co-registration framework, referred to as the Modified SPM. In this approach, random variations are introduced around the optimized solution obtained from the preceding run. Then, each varied value was utilized as a new starting point for re-optimization, and the result yielding the highest mutual information was selected as the final registration result. The registration accuracy was first assessed for each tracer as a whole and then evaluated separately for PET-negative and PET-positive cases to examine the effect of tracer distribution patterns. In addition, registration errors were assessed for PET images without attenuation and scatter correction (uncorrected PET images). For instance, when PET scanners without integrated computed tomography (CT) or MR images are used, uncorrected PET images must be aligned with structural images to generate attenuation correction maps [10, 11]. Finally, we assessed the impact of the repeated optimization process that we introduced into the SPM co-registration framework (Modified SPM).

## Materials and methods

### Participants and PET imaging

Four types of PET tracers were evaluated: two amyloid PET tracers ([^11^C]PiB and [^18^F]florbetapir) and two tau-PET tracers ([^11^C]PBB3 and [^18^F]THK5351). Overall, in this study, 69 participants were enrolled, with the following clinical diagnoses: healthy controls (n = 21), Alzheimer’s disease (n = 20), frontotemporal lobar degeneration (n = 9), progressive supranuclear palsy (n = 8), mild cognitive impairment (n = 4), argyrophilic grain disease (AGD) (n = 4), corticobasal degeneration (n = 2), and posterior cortical atrophy (n = 1).. AGD is fundamentally a neuropathological entity characterized by argyrophilic grain structures and frequently coexists with other neurodegenerative pathologies [12, 13]. Although no internationally established diagnostic criteria for AGD exist, this disease is characterized by morphological asymmetry of the medial temporal lobe [14]. Therefore, in this study, AGD was included as a separate group according to the *Clinical Practice Guideline for Dementia* published by the Japanese Society of Neurology [15] for the purpose of evaluating registration accuracy. The characteristics of the participants are presented in Table 1. This retrospective study was conducted in accordance with the Declaration of Helsinki and its later amendments, and was approved by our institutional review board (approval number K76). The requirement for informed consent was waived due to the retrospective nature of this study.

**Table 1:** Participants characteristics

	[^11^C]PiB	[^18^F]florbetapir	[^11^C]PBB3	[^18^F]THK5351
	n = 25	n = 4	n = 22	n = 18
Age (years)				
average (range)	73 (65, 79)	71 (67, 73)	70 (60, 75)	76 (69, 81)
Male: Female	11: 14	1: 3	14: 8	7: 11
PET-positive: PET-negative	11: 14	2: 2	11: 11	13: 5

PET: positron emission tomography

The administered doses of each PET tracer were as follows: [^11^C]PiB-PET (508 ± 83 MBq), [^18^F]florbetapir (371 ± 16 MBq), [^11^C]PBB3 (618 ± 105 MBq), and [^18^F]THK5351 (186 ± 12 MBq). PET imaging was performed for 20 min starting 40 min post-injection (pi) for [^11^C]PiB-PET; 10 min starting 30 min pi for [^18^F]florbetapir; 30 min starting 30 min pi for [^11^C]PBB3; and 20 min starting 40 min pi for [^18^F]THK5351. All PET data were acquired using a Discovery PET/CT 710 scanner (GE Healthcare, Little Chalfont, PA., USA). Images were reconstructed using three-dimensional ordered-subset expectation maximisation with time-of-flight information (four iterations, 16 subsets). The reconstructed voxel size was 2.0 × 2.0 × 3.27 mm^3^, with a matrix size of 128 × 128 and a field of view of 256 mm. Attenuation, scatter, and random corrections were applied along with a 4-mm Gaussian post-filter. In addition, Uncorrected PET images were prepared for this study before attenuation and scatter corrections. Three-dimensional T1-weighted MR images were acquired using one of the following MRI systems: Vantage Titan (Canon Medical Systems, Otawara, Japan), Discovery MR750 3 T, or Signa HDxt 1.5 T (both from GE Healthcare, Milwaukee, WI, USA).

### Evaluation stratified by PET-negative and PET-positive cases

The acquired PET images were classified as negative or positive by an expert reader (K.I.) with approximately 20 years of experience, based on the visual assessment criteria used in the Japanese Alzheimer’s Disease Neuroimaging Initiative multicenter study [16] or as described in the package insert for amyloid PET imaging. For amyloid PET, scans were classified as positive when the tracer uptake exceeded the adjacent white matter level and extended continuously over at least one gyrus of the cerebral cortex. For tau PET, positivity was defined as tracer uptake in the lateral temporal cortical regions corresponding to Braak NFT stage III or higher [17]. For both amyloid and tau PET, tracer uptake in white matter or deep gray nuclei (e.g., basal ganglia) was not considered in the determination of PET positivity.

### Evaluation using PET images without attenuation and scatter correction

Uncorrected PET images, acquired before attenuation and scatter correction, were prepared separately, whereas the other reconstruction parameters remained the same as those for the corrected PET images. These uncorrected images were used to evaluate registration accuracy separately from corrected PET images, and were analyzed for each tracer without stratifying into negative or positive cases.

### Evaluation method of registration accuracy

**Fig. 1:**
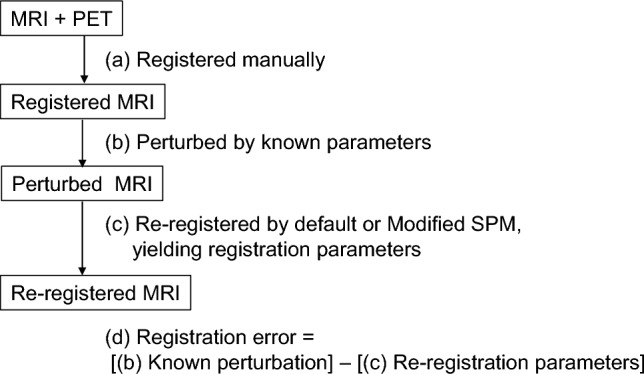
Workflow for evaluating registration accuracy. **a**) Magnetic resonance image (MRI) was manually registered to positron emission tomography (PET) by experts. **b**) Perturbations using randomly generated parameters (standard of truth) were applied to the registered MRI to generate perturbed MRI. **c**) The perturbed MRI was re-registered to the PET using default sStatistical Parametric Mapping (SPM) or Modified SPM, and the resulting re-registration parameters were collected. **d**) The differences between the known perturbation (standard of truth) and re-registration parameters were defined as the registration error

**Fig. 2:**
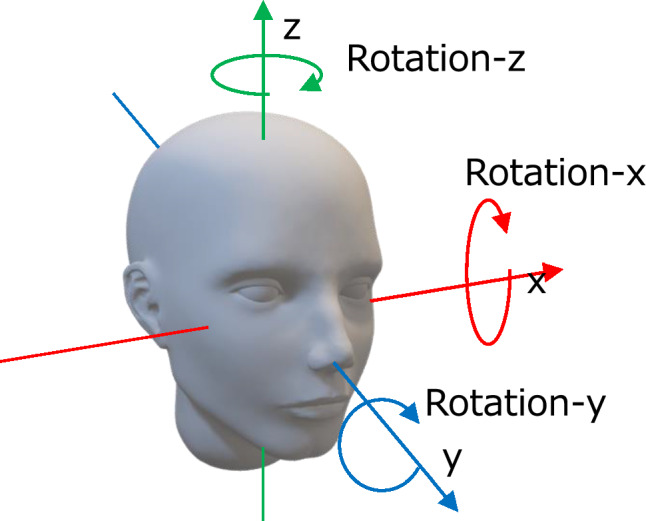
Schematic illustration of translational (x, y, and z) and rotational (rotation-x, rotation-y, and rotation-z) axes used in the registration process; x-axis (left–right), y-axis (anterior–posterior), and z-axis (superior-inferior); rotation-x (pitch, nose tilting up/down), rotation-y (roll, ear up/down), and rotation-z (yaw, nose left/right)

The workflow for the evaluation of registration accuracy evaluation is displayed in Fig. 1. For each participant, fusion images of PET and MR images were generated (Fig. 1a). MR images were manually aligned to the corresponding PET images through consensus by a nuclear medicine physician (M.T.) and technologist (G.A.), with multiple interactive adjustments performed for each case. Manual registration was performed by aligning the anatomical structures across the axial, coronal, and sagittal views, such as the cerebrum, cerebellum, brainstem, and lateral ventricles through visual adjustment of translations and rotations using an in-house viewer capable of voxel-level manipulation Manual registration was performed by aligning the anatomical structures across the axial, coronal, and sagittal views, such as the cerebrum, cerebellum, brainstem, and lateral ventricles. Translation parameters were defined along the x- (left–right), y- (anterior–posterior), and z-axes (superior-inferior), whereas rotation parameters were defined around each corresponding axis (rotation-x, rotation-y, and rotation-z; Fig. 2). Manually registered MR images were intentionally displaced using predefined perturbation parameters to simulate misalignment (Fig. 1b). For each case, 40 perturbation patterns were randomly generated, from a dynamic FDG-PET study, using their actual head motion-derived maximum displacement and rotation ranges [18]: 3.5 mm for x-axis translation, 12.6 mm for z-axis translation, and 5.9 mm for y-axis translation, and 4.1° for rotation-x, 2.1° for rotation-y, and 5.2° for rotation-z. These predefined perturbation parameters were designated as standard truths for the subsequent evaluations. The perturbed MR images were then re-registered to the corresponding PET images (Fig. 1c) using the ‘Coregister’ procedure in SPM12 (Wellcome Centre for Human Neuroimaging, University College London, London, UK) or with a Modified SPM, as described below. The default settings of this procedure, hereafter referred to as ‘default SPM’, were applied: an optimization sampling interval of 4 mm for the initial step and 2 mm for the second and subsequent steps, parameter tolerance settings were left unspecified, and smoothing was applied to the 256 × 256 joint histogram using a 7 × 7 voxel kernel, as required for normalized MI computation. The registration error is defined as the difference between the re-registered parameters and the standard of truth (Fig. 1d).

To the best of our knowledge, no clinically established reference standard for acceptable registration accuracy has been clearly defined. In this study, the voxel size of PET images was 2.0 × 2.0 × 3.27 mm^3^ (x, y, z). Because a voxel represents the smallest unit of spatial resolution in the image, registration errors of less than one voxel were regarded as acceptable. For rotation, < 1.3° was considered acceptable as a 1.3° rotation at the centre of the head, assuming a head diameter of approximately 18 cm [19], corresponds to a displacement of approximately 2 mm along the circumference.

### Modified SPM

In the registration process of the default SPM, six parameters were optimized using Powell’s algorithm [20]. However, the sensitivity of Powell’s method to the initial conditions and its tendency to converge to the local minima may result in inaccurate registration. To address this limitation, we re-implemented the co-registration process in C +  + based on the co-registration framework used in SPM and incorporated a repeated optimization process in the Modified SPM. In this approach, to reduce the likelihood of convergence to local minima, we employed a multi-start strategy wherein optimization was performed multiple times from different initial positions. Specifically, after obtaining an optimal solution, additional starting points were generated by applying random variations to the parameter set, and optimization was repeated for each of these new positions. The magnitude of the random variations applied during initial value generation was set to ± 5%, and the number of iterations was set to 10. Among the 10 repeated optimization runs, the solution that yielded the highest similarity metrics was selected as the final registration result. All other parameters, such as the sampling intervals, tolerance settings, and smoothing, were the same as those used in the default SPM.

### Default vs modified SPM

To compare the two methods, a representative registration error was calculated by assuming the human head to be spherical and computing the distance obtained when the registration error was projected onto the surface of the sphere. Thus, in this study, it is referred to as the spherical surface-based registration error (sRE). Assuming that the registration errors in translation and rotation are denoted as ${t}_{x}, {t}_{y}, {t}_{z}, {r}_{x}, {r}_{y}, {r}_{z}$, the homogeneous transformation matrix ${M}_{err}$ is expressed as follows.

$${M}_{err}=\left(\begin{array}{cccc}1& 0& 0& {t}_{x}\\ 0& 1& 0& {t}_{y}\\ 0& 0& 1& {t}_{z}\\ 0& 0& 0& 1\end{array}\right)\left(\begin{array}{cccc}1& 0& 0& 0\\ 0& \mathit{cos}\left({r}_{x}\right)& \mathit{sin}\left({r}_{x}\right)& 0\\ 0& -\mathit{sin}\left({r}_{x}\right)& \mathit{cos}\left({r}_{x}\right)& 0\\ 0& 0& 0& 1\end{array}\right)$$

1 $$\left(\begin{array}{cccc}\mathit{cos}\left({r}_{y}\right)& 0& \mathit{sin}\left({r}_{y}\right)& 0\\ 0& 1& 0& 0\\ -\mathit{sin}\left({r}_{y}\right)& 0& \mathit{cos}\left({r}_{y}\right)& 0\\ 0& 0& 0& 1\end{array}\right)\left(\begin{array}{cccc}\mathit{cos}\left({r}_{z}\right)& \mathit{sin}\left({r}_{z}\right)& 0& 0\\ -\mathit{sin}\left({r}_{z}\right)& \mathit{cos}\left({r}_{z}\right)& 0& 0\\ 0& 0& 1& 0\\ 0& 0& 0& 1\end{array}\right)$$

Furthermore, in an image space with a voxel size of 2.0 × 2.0 × 3.27 mm^3^, assuming a sphere with a radius of 90 mm to approximate the human head, and denoting the homogeneous coordinates of the surface points as ${x}_{i} (1\le i\le N, N=15110)$, the sRE is defined by the following equation.

2 $$sRE=\frac{1}{N}{\sum }_{i=1}^{N}\Vert M{x}_{i}-{x}_{i}\Vert $$

This metric enables the quantitative evaluation of the combined effects of the six registration parameters, translations, and rotations.

### Verification of the impact of additional processes in the modified SPM

The Modified SPM was implemented in C +  + , replicating the co-registration framework used in the default SPM and repeated optimization process to facilitate future program revisions. To exclude any potential impact of the C +  + implementation on the results, we conducted a further comparison. A digital human brain phantom simulating actual FDG distribution, the BigBrain phantom [21], was used for verification. As this PET phantom was created from MR images, it precisely matched the boundaries of both the PET and MR images. The PET and MRI data were resampled to a voxel size of 2.0 × 2.0 × 3.27 mm^3^ and smoothed with a 4-mm Gaussian post-filter. The MR images were then transformed using the 40 patterns of perturbed parameters described above and re-registered to PET using each of the registration methods as follows.

1. Default SPM,
2. Modified SPM without the repeated optimization process (replicating the default SPM in C + +), and.
3. Modified SPM.

For each registration, sRE was calculated.

### Impact of registration error on volume-of-interest analysis

A widely used volume-of-interest (VOI) template, AAL atlas, consisting of 68 VOIs in PMOD version 4.3 (PMOD Technologies LLC, Zurich, Switzerland), was morphologically applied to the MRI digital phantom, which was resliced to match the PET voxel size (2.0 × 2.0 × 3.27 mm^3^) used in this study. The resliced and shape-matched VOI template was then perturbed using the 2,760 parameter sets to simulate 2,760 patterns of misregistration generated in this study. To evaluate voxel-level consistency before and after perturbation, we calculated the proportion of voxels that remained within the same VOI after perturbation. This analysis was performed according to the magnitude of sRE, categorized as 0–2 mm, 2–4 mm, and 4–6 mm.

### Differences in registration accuracy between 1.5 T and 3 T T1-weighted MRI

To evaluate the effect of MRI field strength on registration accuracy, the sRE values for each tracer were calculated separately for cases using 1.5 T or 3 T T1-weighted MRI. The numbers of cases acquired with 1.5 or 3 T MRI were 5 and 20 for [^11^C]PiB, 2 and 2 for [^1^⁸F]florbetapir, 17 and 5 for [^11^C]PBB3, and 4 and 16 for [^1^⁸F]THK5351, respectively.

### Reproducibility of the Modified SPM method

To evaluate the reproducibility of the Modified SPM, we used a digital human brain phantom (BigBrain phantom), which was resliced to match the PET voxel size used in this study (2.0 × 2.0 × 3.27 mm^3^). The phantom data were perturbed using the same 40 parameter patterns applied in the registration experiments. Each perturbed phantom dataset was reregistered to the reference using the Modified SPM, and this procedure was repeated 10 times per perturbation pattern. Reproducibility was assessed by calculating the standard deviation of the resulting registration parameters across the 10 repeated runs for each perturbation pattern.

### Statistical analysis

Forty patterns derived from six perturbation parameters were analyzed for each of the 69 participants, resulting in 2,760 patterns. The absolute registration errors are expressed as average values, standard deviations, and maximum values. Statistical comparisons of the registration errors were performed using Welch’s t-tests. An F-test was performed to compare the variances in the registration error values. Statistical significance was set at *p* < 0.05.

## Results

**Fig. 3:**
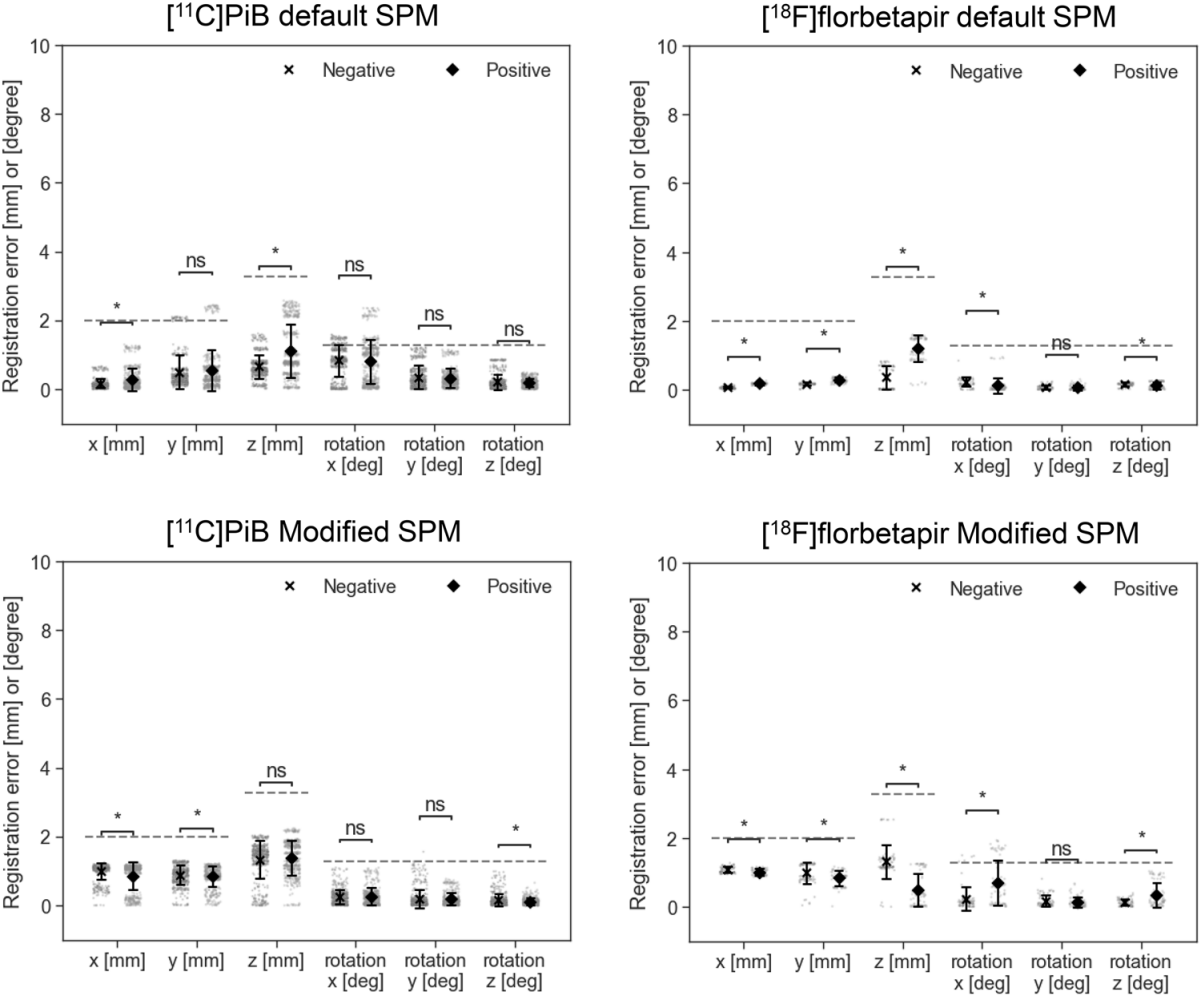
Registration errors stratified by positron emission tomography (PET)-negative and PET-positive cases of amyloid-PET ([^11^C]PiB and [.^18^F]florbetapir) using the default (upper row) or Modified statistical parametric mapping (SPM) (lower row). All PET images shown are attenuation and scatter-corrected. The dashed lines are corresponding to the size of one voxel. Bars represent mean ± standard deviation. Each plot represents a single registration run. *p < 0.05 (Welch’s t-test)

**Fig. 4:**
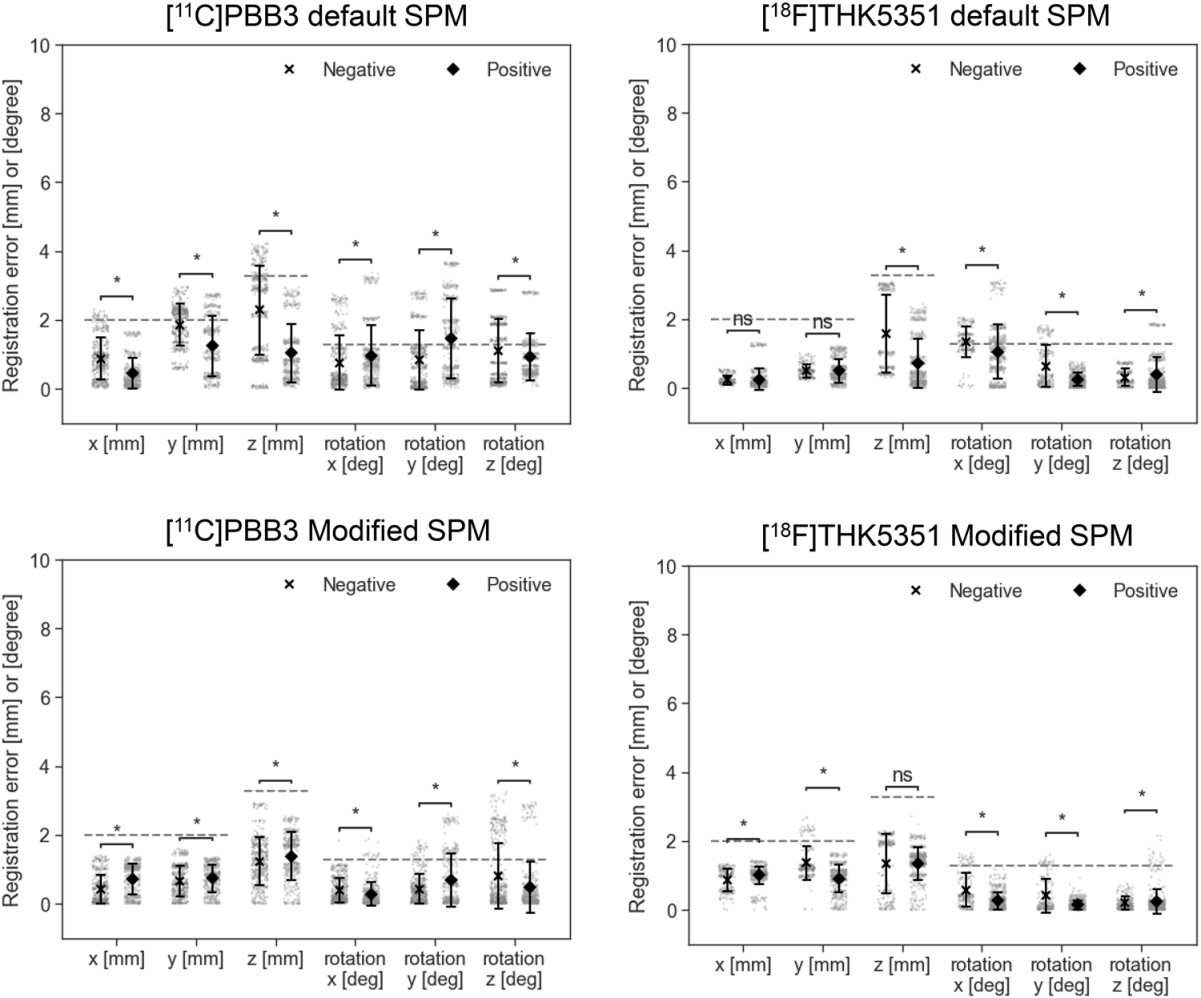
Registration errors stratified by positron emission tomography (PET)-negative and PET-positive cases of tau-PET images ([^11^C]PBB3 and [.^18^F]THK5351) using the default (upper row) or Modified statistical parametric mapping (SPM) (lower row). All PET images shown are attenuation and scatter-corrected. The dashed lines are corresponding to the size of one voxel. Bars represent mean ± standard deviation. Each plot represents a single registration run. *p < 0.05 (Welch’s t-test)

The registration errors across the translational and rotational axes, as well as the tracer type, were evaluated using corrected PET images and are summarized in Table 2. A total of 2,760 registration runs were performed, corresponding to 40 perturbation patterns per case, across 69 cases: [11C]PiB-PET (25 cases). [18F]florbetapir (4 cases), [11C]PBB3 (22 cases), and [18F]THK5351(18 cases). The average registration errors were less than the size of one voxel (2.0 × 2.0 × 3.27 mm^3^) or corresponding rotation angle of 1.3°, and were, therefore, considered acceptable with both the default and Modified SPM. In addition, the PET images were further stratified into PET-negative and PET-positive cases for amyloid and tau-PET, and the registration errors are presented in Figs. 3 and 4, respectively. The detailed numerical values are provided in Supplementary Table 1. Using the default SPM, the average registration errors, when stratified by PET-negative and PET-positive cases, were less than the size of one voxel, except for rotation-y of [^11^C]PBB-PET positive cases and for rotation-x of [^18^F]THK5351-PET negative cases, but these were less than the size of one voxel with the Modified SPM. In amyloid-PET images, PET-positive cases tended to have higher registration errors than those of the PET-negative cases with the default SPM.

**Table 2:** Summary of registration errors

Default SPM	x		y		z			x		y		z	
	Ave (SD)	Max	Ave (SD)	Max	Ave (SD)		Max	Ave (SD)	Max	Ave (SD)	Max	Ave (SD)	Max
[^11^C]PiB	0.2(0.3)	1.3	0.5(0.5)	2.5	0.9(0.6)		2.6	0.8(0.6)	2.4	0.3(0.3)	1.2	0.2(0.2)	0.9
[^18^F]florbetapir	0.1(0.0)	0.3	0.2(0.1)	0.4	0.8(0.6)	1.6		0.2(0.2)	0.9	0.1(0.1)	0.3	0.1(0.1)	0.3
[^11^C]PBB3	0.7(0.6)	2.3	1.6(0.8)	3.0	1.7(1.3)		4.2	0.9(0.8)	3.4	1.1(1.1)	3.7	1.0(0.8)	2.9
[^18^F]THK5351	0.2(0.3)	1.3	0.5(0.3)	1.2	0.9(0.9)		3.1	1.1(0.7)	3.1	0.4(0.4)	1.8	0.4(0.4)	1.9
	Translation [mm]							Rotation [degree]					
Modified SPM	x		y		z			x		y		z	
	Ave (SD)	Max	Ave (SD)	Max	Ave (SD)		Max	Ave (SD)	Max	Ave (SD)	Max	Ave (SD)	Max
[^11^C]PiB	0.9(0.3)	1.2	0.9(0.3)	1.3	1.3(0.5)		2.2	0.2(0.2)	1.1	0.2(0.2)	1.6	0.1(0.1)	1.0
[^18^F]florbetapir	1.0(0.1)	1.3	0.9(0.3)	1.3	0.9(0.6)		2.5	0.5(0.6)	1.9	0.1(0.2)	0.8	0.2(0.3)	1.0
[^11^C]PBB3	0.6(0.5)	1.4	0.7(0.4)	1.6	1.3(0.7)		3.3	0.3(0.4)	1.9	0.6(0.6)	2.6	0.6(0.9)	3.2
[^18^F]THK5351	1.0(0.3)	1.4	1.0(0.5)	2.7	1.3(0.6)		2.7	0.3(0.4)	1.6	0.2(0.3)	1.6	0.2(0.3)	2.1

SPM: statistical parametric mapping, SD: standard deviation

**Fig. 5:**
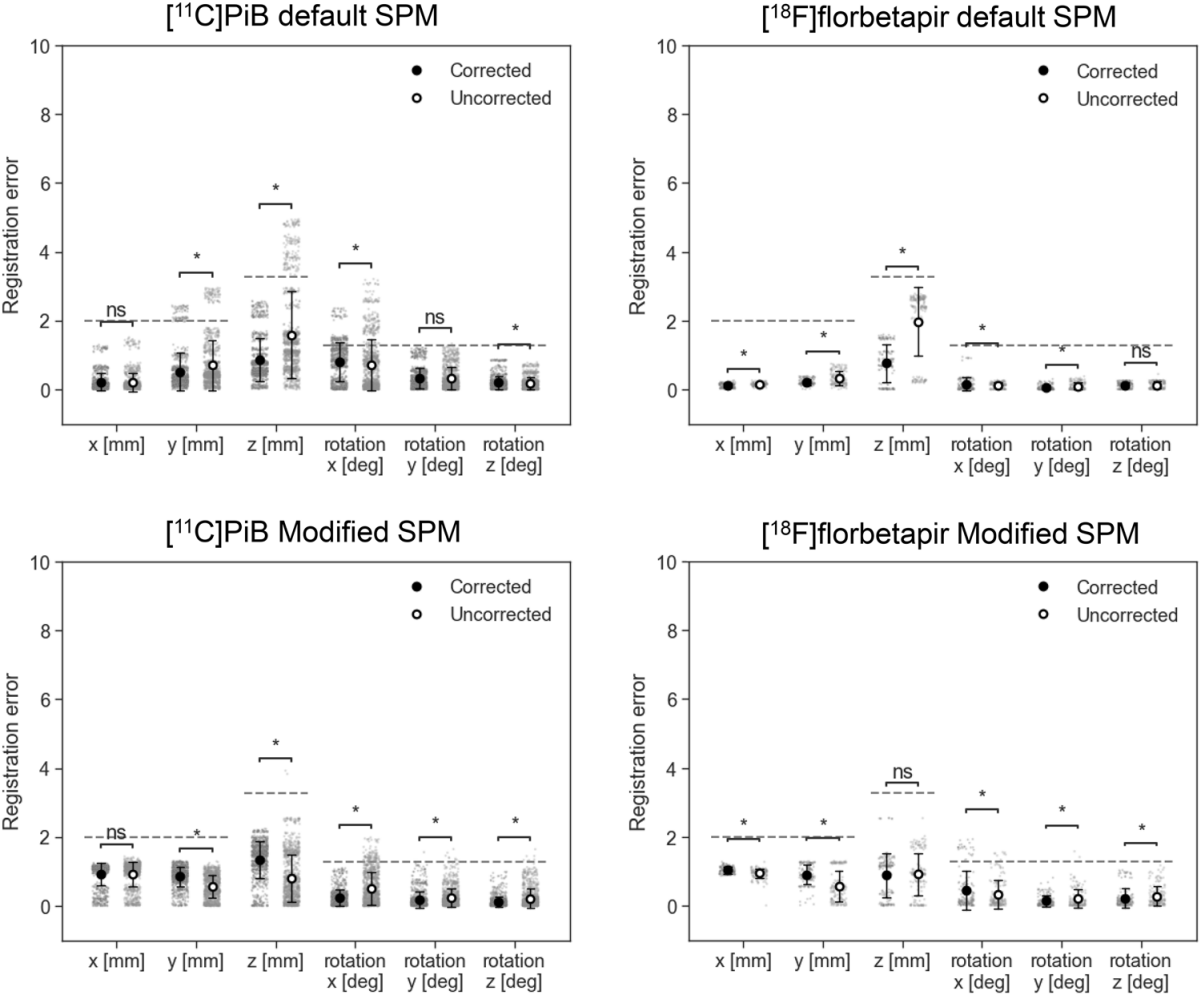
Registration errors for positron emission tomography (PET) images with and without attenuation- and scatter-correction (corrected and uncorrected PET images, respectively) using the default (upper row) or Modified statistical parametric mapping (SPM) (lower row). The dashed lines are corresponding to the size of one voxel. Bars represent mean ± standard deviation. Each plot represents a single registration run. *p < 0.05 (Welch’s t-test)

**Fig. 6:**
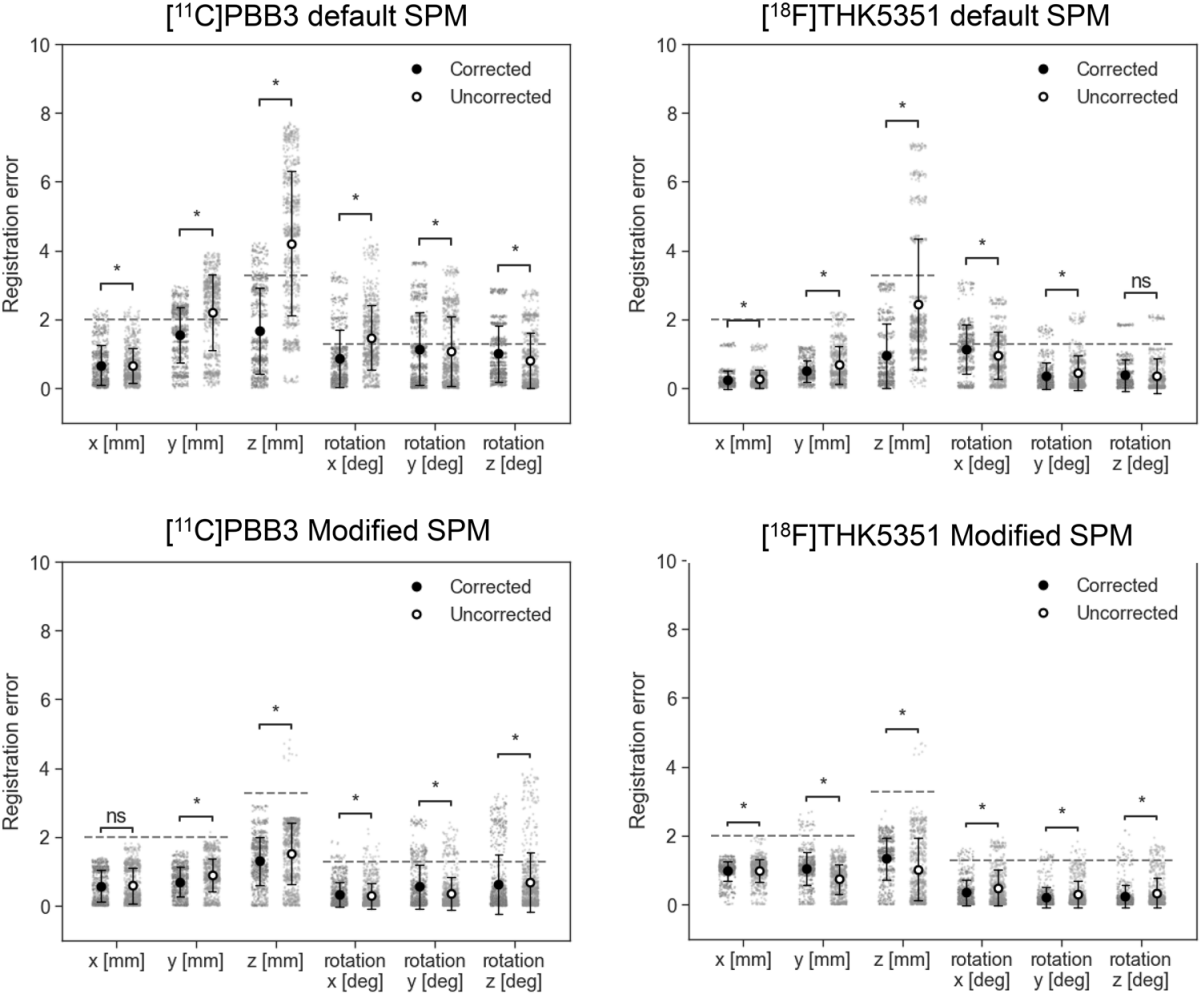
Registration errors for positron emission tomography (PET) images with and without attenuation- and scatter-correction (corrected and uncorrected PET images, respectively) using the default (upper row) or Modified statistical parametric mapping (SPM) (lower row). The dashed lines are corresponding to the size of one voxel. Bars represent mean ± standard deviation. Each plot represents a single registration run. *p < 0.05 (Welch’s t-test)

The registration errors for the corrected and uncorrected PET images are shown in Figs. 5 and 6, and detailed numerical values are presented in Supplementary Table 2. Uncorrected PET images tended to have higher registration errors than that of corrected PET images, and average errors exceeding the size of one voxel were observed in the uncorrected [^11^C]PBB3 PET images with the default SPM. Using the Modified SPM, these errors were reduced to less than one voxel.

**Fig. 7:**
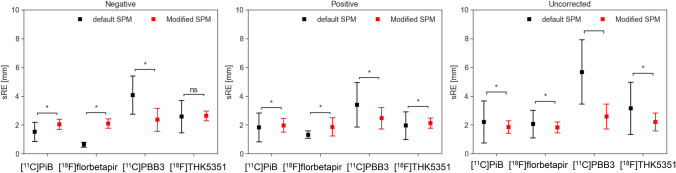
Comparisons between the default and Modified statistical parametric mapping (SPM) in the representative registration error, referred to as sRE (spherical-surface-based registration error), which represents the corresponding displacement on the surface assuming the head is spherical. These comparisons were conducted for each tracer, stratified by positron emission tomography (PET)-negative and PET-positive cases in corrected PET images. Comparisons in uncorrected PET images were not stratified by PET-negative and PET-positive cases. Bars represent mean ± standard deviation. *p < 0.05 (Welch’s t-test). An F-test revealed significant differences in variance, and the Modified SPM yielded significantly smaller variances than the default SPM for all tracers except [^1^⁸F]florbetapir

Comparisons of the sRE between the default and Modified SPM are shown in Fig. 7. The Modified SPM decreased the sRE in both negative and positive tau-PET images, except for [^18^F]THK5351-PET negative cases, but increased the sRE in amyloid-PET images. For the uncorrected images, the Modified SPM reduced the sRE for all tracers. The variance of the sRE was significantly reduced in the Modified SPM for both PET-negative and PET-positive cases for all tracers, except [^18^F] florbetapir.

**Fig. 8:**
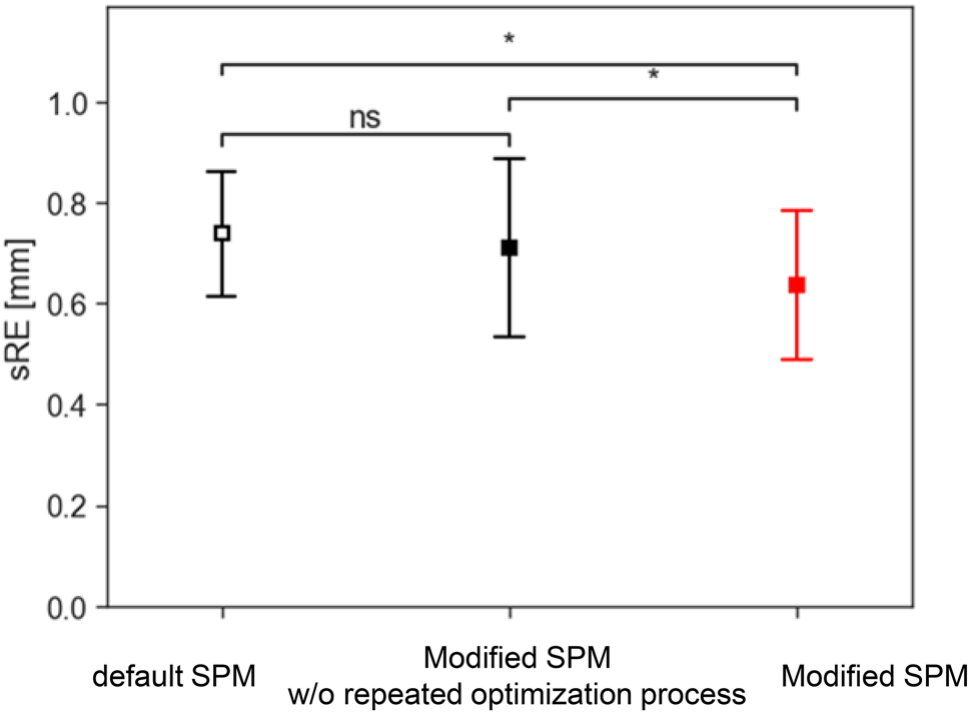
Comparisons of representative registration error (spherical-surface-based registration error; sRE) among the default statistical parametric mapping (SPM), Modified SPM without the repeated optimization process, and Modified SPM. Bars represent mean ± standard deviation. *p < 0.05 (Welch’s t-test)

The results of the sRE comparison using the default SPM, Modified SPM without the repeated optimization process, and the Modified SPM are presented in Fig. 8. No significant difference was observed between the default and Modified SPM without the additional processes, indicating that the implementation of the SPM co-registration framework in C +  + had no effect on these results. The sRE was significantly lower with the Modified SPM than with the other methods (vs the default SPM, p < 0.001; vs the Modified SPM without the optimization process, p = 0.045).

**Fig. 9:**
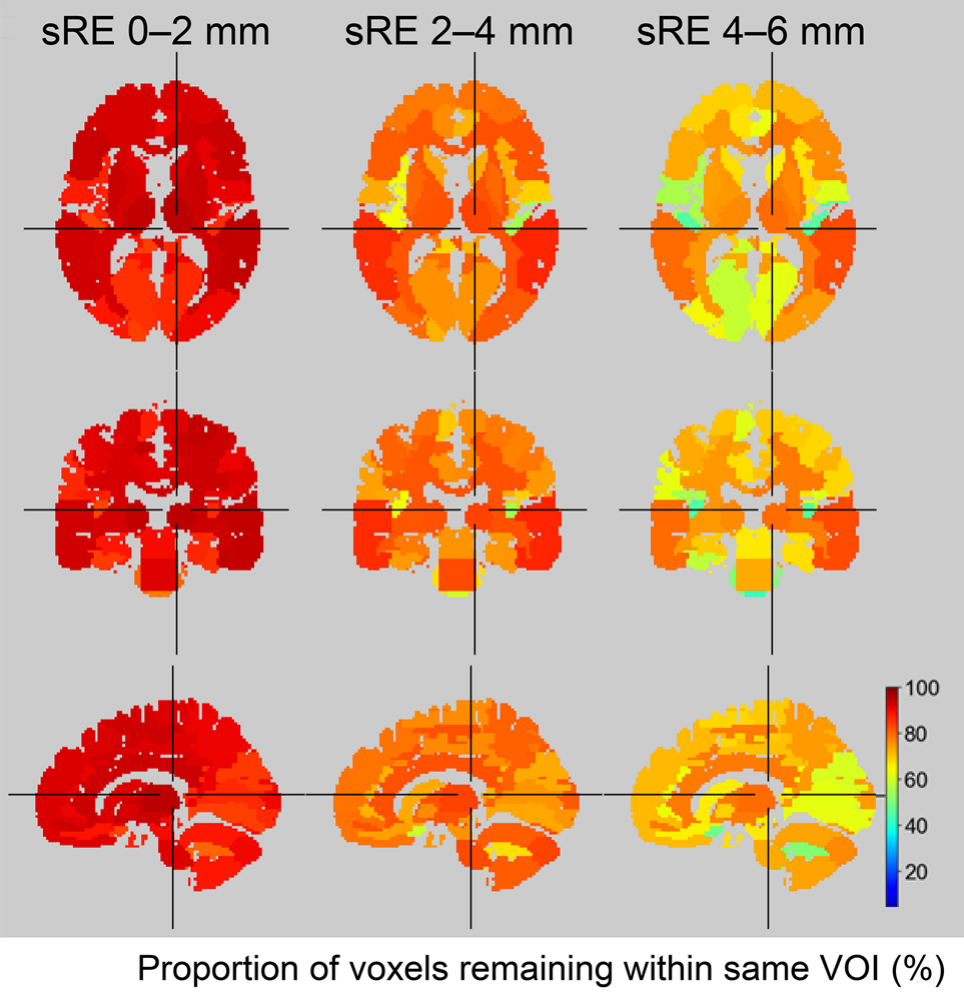
Representative heat maps showing the percentage of voxels that remained within the same voxel-of-interest (VOI) after perturbation. sRE denotes the spherical surface-based registration error, as defined by Eqs. (1) and (2) in the Materials and Methods section

The proportions of voxels that remained within the same VOI were 89 ± 10%, 75 ± 7%, and 66 ± 7% (mean ± SD) for sRE of 0–2 mm, 2–4 mm, and 4–6 mm, respectively. The spatial distribution of voxel consistency is shown in Fig. 9. In the VOIs showing the minimum consistency was 78% for sRE of 0–2 mm, whereas it declined to 55% and 42% for sRE of 2–4 mm and 4–6 mm, respectively.

**Fig. 10:**
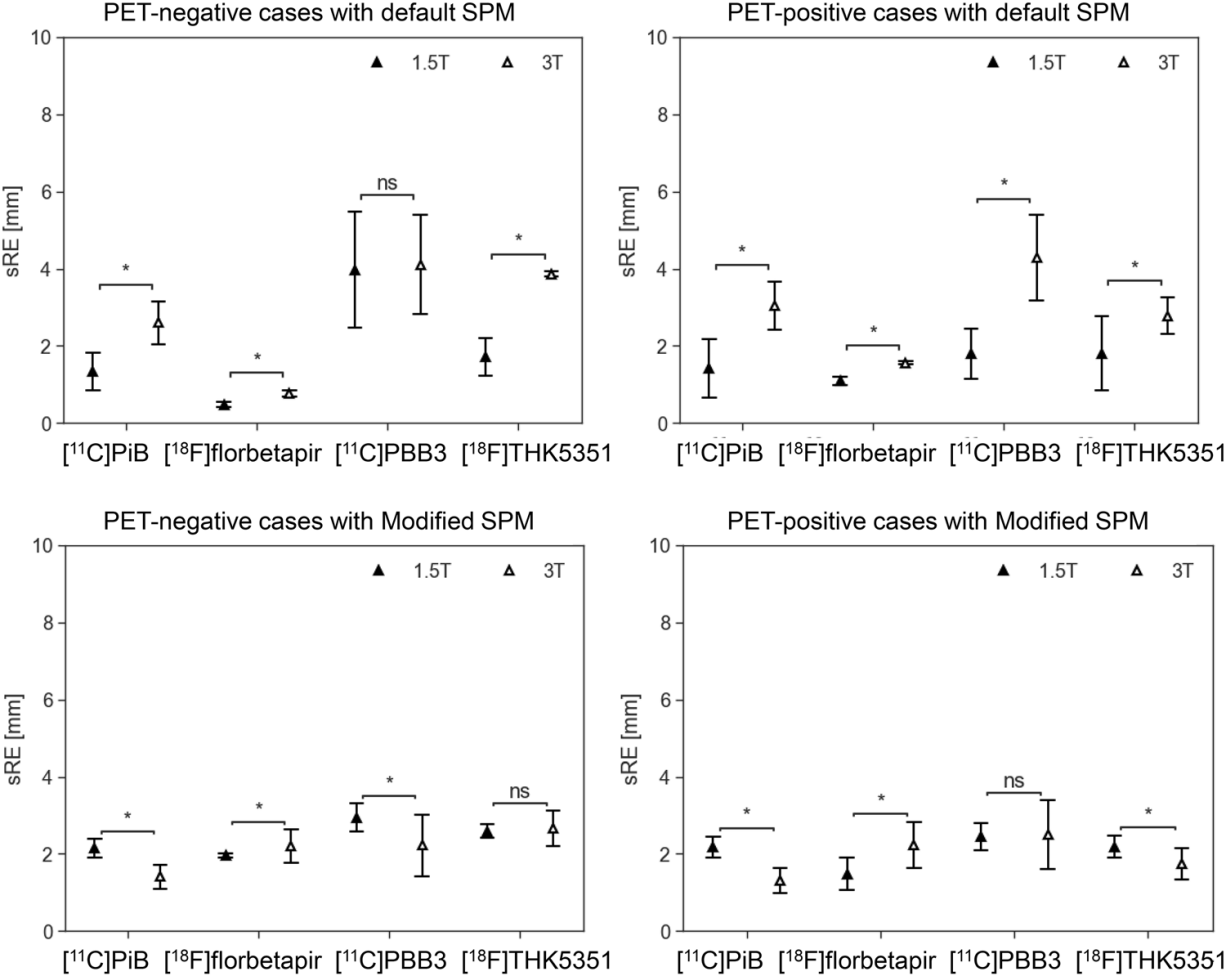
Comparisons of representative registration error (spherical-surface-based registration error; sRE) according to whether 1.5 T or 3 T T1-weighted MRIwas used. Bars represent mean ± standard deviation. *p < 0.05 (Welch’s t-test)

The results of the sRE comparison according to 1.5 T or 3 T T1-weighted MRI used are shown in Fig. 10. With the default SPM, registration errors tended to be larger when using 3 T MRI compared with 1.5 T MRI. In contrast, with the Modified SPM, registration errors were smaller with 3 T MRI than with 1.5 T MRI, except for [^18^F] florbetapir PET.

For the reproducibility experiment, the mean standard deviation of the registration results across all perturbation patterns was 0.025 mm.

## Discussion

Registration errors between the MR and PET images were evaluated using recently available amyloid and tau tracers: [^11^C]PiB, [^18^F]florbetapir, [^11^C]PBB3, and [^18^F]THK5351. The average errors across the tracers were acceptable and less than one voxel. When stratified by PET-positive and PET-negative cases, average registration errors exceeding one voxel were observed in the [^11^C]PBB3-positive and [^18^F]THK5351-negative PET images. However, the use of the Modified SPM reduced these errors to less than one voxel. The Modified SPM was also effective for uncorrected PET images, indicating its utility in registering images with a low statistical dependency between image intensity distributions, such as tau and uncorrected PET images.

A previous study using FDG-PET reported the maximum errors of up to 3 mm in translation and 4° in rotation [1]. In the present study, the results for [^11^C]PiB and [^18^F]florbetapir-PET were within these ranges, whereas those for the tau-PET tracers [^11^C]PBB3 and [^18^F]THK5351 were not, suggesting that registration accuracy may vary depending on the specific tracer used.

**Fig. 11:**
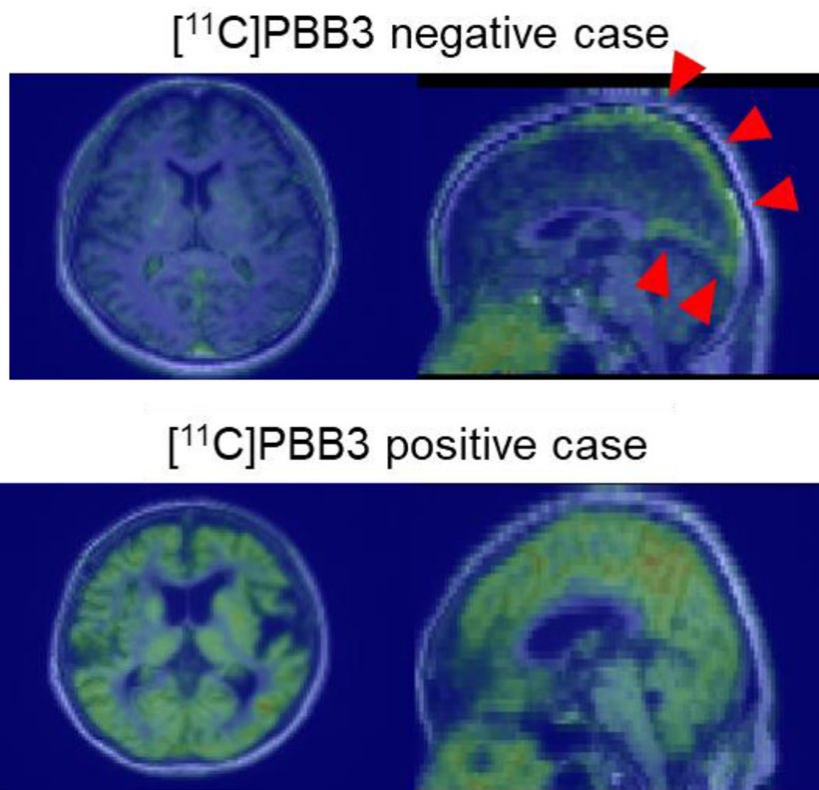
Representative [^11^C]PBB3 positron emission tomography (PET) images. The upper row images show a negative image, with an arrowhead indicating [^11^C]PBB3 retention in the venous sinus. The lower row images present a positive case, demonstrating specific tracer accumulation in the cerebral cortex

Errors along the z-axis (translation-z) were the most pronounced, corresponding to head movements in the up/down direction, particularly in [^11^C]PBB3 PET-negative cases. A possible explanation for the increased error in this direction is the retention of [^11^C]PBB3 in the venous sinuses (Fig. 11). In these [^11^C]PBB3-PET images, particularly in negative cases, the signal intensity in the brain parenchyma differed from that in the corresponding MR images. The combination of high signal intensity in the venous sinuses and low signal intensity in the parenchyma may reduce the anatomical similarity between PET and MRI, leading to diminished registration accuracy. The relatively large errors observed along the z-axis across tracers may be partly attributable to the larger voxel size in the z direction, which reduces the statistical dependence of the intensity distribution between PET and MRI during registration. In addition, Ikari et al. reported that head motion tends to be greater along the z-axis (12.6 mm at maximum) compared with the y- and x-axes (5.9 mm and 3.5 mm, respectively) [18], suggesting that this directional tendency of patient motion may also have contributed to the increased z-axis misregistration.

The MI cost function tends to reduce performance when the statistical dependence of intensity distributions is weak, and this tendency was suggested in the present study for tracers such as PBB3 in this study. As alternative cost functions, we considered approaches such as the Modality Independent Neighborhood Descriptor [22] and deep-learning–based feature extraction approaches. Although these methods offer advantages in capturing local image features, global consistency is more important than local structural correspondence when registering rigid anatomical structures such as the human brain. Therefore, MI, which evaluates intensity relationships across the entire image, is likely more suitable. A hybrid approach that combines MI with deep-learning-based local feature extraction may improve accuracy and robustness.

When using a dedicated PET scanner without an accompanying CT or MRI [10, 11], PET images before attenuation and scatter correction must be registered to CT or MR images. The uncorrected PET images exhibited a tendency for larger registration errors than the corrected PET images (Figs. 5 and 6); however, the Modified SPM helped reduce these errors. Uncorrected PET images exhibit increased noise and heterogeneous tissue contrast, particularly enhancing artefacts in extracerebral regions, resulting in a more multimodal (i.e., containing multiple peaks) cost function, which may increase the likelihood of convergence to the local minima. The Modified SPM improved the registration accuracy, presumably by enhancing convergence to the global optimum using a multi-start strategy wherein the registration process is repeated multiple times from different starting points around the previously converged solution.

To assess the potential impact of registration error on the VOI-based analysis, we examined voxel-level correspondence using a digital brain phantom. Misregistration of approximately one voxel (≈2 mm) or greater on average substantially degrades VOI correspondence and may compromise the reliability of regional quantitative analysis. When the average sRE was within 2 mm on the assumed spherical surface of the human brain, up to 22% of voxels were incorrectly assigned to different anatomical regions. The impact was more pronounced in small VOIs, such as the Heschl gyrus and amygdala, where only approximately 60% of voxels remained within the correct VOI when the misregistration exceeded one voxel (i.e., sRE of 2–4 mm). In amyloid and tau PET imaging, visual assessment of tracer uptake in the cerebral cortex relative to white matter is the standard diagnostic approach. Given that cortical thickness is typically on the order of 3–5 mm, misregistration exceeding one voxel may substantially compromise both quantitative accuracy and visual interpretability. The frequency of cases in which the maximum error exceeds one voxel is summarized in Table 3. The use of the Modified SPM reduced this frequency from 79 to 30%. These findings indicated that improved registration accuracy was achieved with the Modified SPM, which is particularly advantageous for quantitative analysis in small brain regions.

**Table 3:** Frequency of cases where the maximum error exceeds one voxel

default SPM	Negative			Positive	
	Percentage (%) of cases with				
	> 1 voxel	> 2 voxels		> 1 voxel	> 2 voxels
[^11^C]PiB	21	0		25	0
[^18^F]florbetapir	0	0		0	0
[^11^C]PBB3	73	22		64	42
[^18^F]THK5351	79	0		44	8
Modified SPM	Negative			Positive	
	Percentage (%) of cases with				
	> 1 voxel	> 2 voxels		> 1 voxel	> 2 voxels
[^11^C]PiB	0	0		0	0
[^18^F]florbetapir	6	0		25	0
[^11^C]PBB3	30	4		30	6
[^18^F]THK5351	30	0		5	0

SPM: statistical parametric mapping

The effect of MRI field strength on registration accuracy showed opposite trends between the default SPM and Modified SPM methods. The reduced accuracy observed with 3 T MRI using the default SPM is likely attributable to its higher signal-to-noise ratio (SNR), which preserves fine anatomical structures that are not represented in amyloid or tau PET images. These fine-scale structural discrepancies may increase the likelihood that the mutual information–based cost function becomes trapped in local minima. Conversely, the lower SNR of 1.5 T MRI may have an effect similar to image smoothing, attenuating fine structural differences and thereby facilitating more robust multimodal registration. The accuracy gap between 1.5 T and 3 T MRI was reduced using the modified SPM, suggesting that the multi-start strategy mitigates convergence to local minima and stabilizes the registration process across different MRI field strengths.

The reproducibility assessment using the digital human brain phantom demonstrated that the Modified SPM yields highly stable registration results, with an average standard deviation of only 0.025 mm across repeated runs. Given that this variability is negligible relative to the voxel size, the convergence behavior of the Modified SPM can be considered stable and reproducible.

The introduction of a multi-start strategy was expected to increase computational time. The Modified SPM was implemented in C +  + for efficiency. In practice, the processing time was 25.9 ± 6.2 s for the default SPM and 18.6 ± 5.7 s for the Modified SPM, indicating a significantly shorter runtime for the Modified SPM. Although the absolute processing time depends on the computing environment, these results suggest that the additional optimization steps do not impose a substantial computational burden.

We also considered other commonly used PET–MRI registration tools, including NiftyReg [23], VINCI [24], FSL (FMRIB Software Library) [25], and ANTs (Advanced Normalization Tools) [26]. These tools similarly employ mutual information–based optimization for multimodal registration; therefore, some challenges observed in this study, such as reduced registration accuracy for tau PET images, are likely common across these methods. However, as demonstrated by Markiewicz et al., implementation differences among software packages can substantially influence registration performance, and detailed trends may vary between tools [2]. In particular, FSL has been reported to be less suitable for PET applications, suggesting that similar limitations may also apply to tau PET. ANTs has been shown to achieve higher registration accuracy than SPM [27], potentially due to its gradient-based optimization strategy; however, that evaluation was limited to intra-modality MRI registration. The multi-start strategy adopted in the Modified SPM, which is also implemented by default in FSL to improve convergence, may therefore be beneficial in other registration frameworks as well.

We have some limitations in this study. One is the use of manual registration as the standard of truth. However, there is no established ground truth for the registration of separately acquired images. Therefore, we carefully performed manual registration on a case-by-case basis as a practical surrogate for standards of truth. Tthe Modified SPM consistently demonstrated stable convergence across both digital phantom data, in which PET and MRI images are intrinsically matched, and clinical datasets, suggesting that the manual registration did not introduce systematic bias. The reproducibility of manual registration was assessed only by visual inspection. Given that the ability of visual inspection to detect PET–MRI misregistration has been reported to be limited to approximately 2–3 mm [1], this may represent a methodological limitation of this study. Additionally, because the voxel size of the reconstructed PET images was 2.0 × 2.0 × 3.27 mm^3^, we focused our evaluation on the size of one voxel, considering its clinical relevance.

Another limitation of this study is that the precise reasons for the observed differences among the registration methods could not be fully elucidated. The sRE values for amyloid PET ([^11^C]PiB, [^18^F]florbetapir) were larger with the Modified SPM than with the default SPM (Fig. 7). However, because the difference was small (within one voxel), it is unlikely to represent a systematic effect of the multi-start strategy. Rather, it may be attributable to minor implementation-related numerical differences in the C +  + version, such as convergence criteria or interpolation schemes.

In conclusion, registration between PET and MR images is crucial in PET studies; however, the accuracy varies depending on the distribution characteristics of the PET tracer, as observed in tau-PET images. While the registration errors were within acceptable limits, relatively large errors were found in the tau-PET and PET images without attenuation and scatter correction. The repeated optimization process incorporated into the Modified SPM may, therefore, be beneficial for improving the registration accuracy when using specific tracers or uncorrected PET images.

## Supplementary Information

Below is the link to the electronic supplementary material.

Supplementary file1 (DOCX 42 KB)

## Data Availability

The datasets used and analyzed in the current study are available from the corresponding author upon reasonable request.
