# Supplementary material for: Registration accuracy of amyloid/tau-PET to brain MRI using modified SPM method
Source: Ann Nucl Med. 2026 Feb 4;40(5):584–96. doi: 10.1007/s12149-026-02159-3 (PMC13124865; doi:10.1007/s12149-026-02159-3)
Supplement: Supplementary file 1 — Supplementary file1 (DOCX 42 KB) [file 12149_2026_2159_MOESM1_ESM.docx]

*Annals of Nuclear Medicine*

**Registration Accuracy of Amyloid/Tau-PET to Brain MRI using Modified SPM Method**

Yuma Iwao,^1^ Go Akamatsu,^1^ Muneyuki Sakata,^2^ Kei Wagatsuma,^2,3^ Kenji Ishii,^2^ Taiga Yamaya^1^, and Miwako Takahashi,^1*^

**Corresponding author:**

Miwako Takahashi

4-9-1 Anagawa, Inage-ku, Chiba-shi, Chiba, 263-8555, Japan

E-mail: takahashi.miwako@qst.go.jp

Supplementary Table 1. Registration errors stratified by positron emission tomography (PET)-negative and PET-positive amyloid or tau PET cases

|  | Translation [mm] | | | | | | | |  | Rotation [degree] | | | | | | | |
| --- | --- | --- | --- | --- | --- | --- | --- | --- | --- | --- | --- | --- | --- | --- | --- | --- | --- |
| default  SPM | x | |  | y | |  | z | |  | x | |  | y | |  | z | |
|  | Ave (SD) | Max |  | Ave (SD) | Max |  | Ave (SD) | Max |  | Ave (SD) | Max |  | Ave (SD) | Max |  | Ave (SD) | Max |
| [^11^C]PiB |  |  |  |  |  |  |  |  |  |  |  |  |  |  |  |  |  |
| Negative | 0.2(0.1) | 0.7 |  | 0.5(0.5) | 2.1 |  | 0.6(0.3) | 1.6 |  | 0.8(0.5) | 1.6 |  | 0.3(0.3) | 1.2 |  | 0.2(0.2) | 0.9 |
| Positive | 0.3(0.3) | 1.3 |  | 0.5(0.6) | 2.5 |  | 1.1(0.8) | 2.6 |  | 0.8(0.6) | 2.4 |  | 0.3(0.3) | 1.1 |  | 0.2(0.1) | 0.5 |
| [^18^F]florbetapir |  |  |  |  |  |  |  |  |  |  |  |  |  |  |  |  |  |
| Negative | 0.1(0.0) | 0.1 |  | 0.2(0.0) | 0.2 |  | 0.4(0.3) | 0.8 |  | 0.2(0.1) | 0.8 |  | 0.1(0.1) | 0.2 |  | 0.1(0.0) | 0.2 |
| Positive | 0.2(0.0) | 0.3 |  | 0.3(0.1) | 0.4 |  | 1.2(0.4) | 1.6 |  | 0.1(0.2) | 0.9 |  | 0.1(0.1) | 0.3 |  | 0.1(0.1) | 0.3 |
| [^11^C]PBB3 |  |  |  |  |  |  |  |  |  |  |  |  |  |  |  |  |  |
| Negative | 0.9(0.6) | 2.3 |  | 1.9(0.6) | 3.0 |  | 2.3(1.3) | 4.2 |  | 0.8(0.8) | 2.7 |  | 0.8(0.9) | 2.8 |  | 1.1(0.9) | 2.9 |
| Positive | 0.4(0.5) | 1.7 |  | 1.2(0.9) | 2.8 |  | 1.0(0.8) | 2.9 |  | 1.0(0.9) | 3.4 |  | 1.5(1.2) | 3.7 |  | 0.9(0.7) | 2.8 |
| [^18^F]THK5351 |  |  |  |  |  |  |  |  |  |  |  |  |  |  |  |  |  |
| Negative | 0.2(0.1) | 0.5 |  | 0.5(0.2) | 1.0 |  | 1.6(1.1) | 3.1 |  | 1.3(0.4) | 2.0 |  | 0.6(0.6) | 1.8 |  | 0.3(0.2) | 0.8 |
| Positive | 0.2(0.3) | 1.3 |  | 0.5(0.3) | 1.2 |  | 0.7(0.7) | 2.5 |  | 1.1(0.8) | 3.1 |  | 0.3(0.2) | 0.8 |  | 0.4(0.5) | 1.9 |
|  |  |  |  |  |  |  |  |  |  |  |  |  |  |  |  |  |  |
|  |  |  |  |  |  |  |  |  |  |  |  |  |  |  |  |  |  |
|  | Translation [mm] | | | | | | | |  | Rotation [degree] | | | | | | | |
| Modified  SPM | x | |  | y | |  | z | |  | x | |  | y | |  | z | |
|  | Ave (SD) | Max |  | Ave (SD) | Max |  | Ave (SD) | Max |  | Ave (SD) | Max |  | Ave (SD) | Max |  | Ave (SD) | Max |
| [^11^C]PiB |  |  |  |  |  |  |  |  |  |  |  |  |  |  |  |  |  |
| Negative | 1.0(0.2) | 1.2 |  | 0.9(0.3) | 1.3 |  | 1.3(0.5) | 2.0 |  | 0.2(0.2) | 1.1 |  | 0.2(0.3) | 1.6 |  | 0.1(0.2) | 1.0 |
| Positive | 0.8(0.4) | 1.2 |  | 0.8(0.3) | 1.2 |  | 1.4(0.5) | 2.2 |  | 0.3(0.2) | 1.1 |  | 0.2(0.2) | 0.8 |  | 0.1(0.1) | 0.7 |
| [^18^F]florbetapir |  |  |  |  |  |  |  |  |  |  |  |  |  |  |  |  |  |
| Negative | 1.1(0.1) | 1.3 |  | 1.0(0.3) | 1.3 |  | 1.3(0.5) | 2.5 |  | 0.2(0.3) | 1.5 |  | 0.2(0.2) | 0.8 |  | 0.1(0.1) | 0.4 |
| Positive | 1.0(0.1) | 1.2 |  | 0.8(0.2) | 1.2 |  | 0.5(0.5) | 1.3 |  | 0.7(0.6) | 1.9 |  | 0.2(0.2) | 0.7 |  | 0.3(0.4) | 1.0 |
| [^11^C]PBB3 |  |  |  |  |  |  |  |  |  |  |  |  |  |  |  |  |  |
| Negative | 0.4(0.4) | 1.4 |  | 0.7(0.4) | 1.6 |  | 1.2(0.7) | 2.9 |  | 0.4(0.4) | 1.8 |  | 0.4(0.4) | 1.8 |  | 0.8(0.9) | 3.2 |
| Positive | 0.7(0.4) | 1.4 |  | 0.7(0.4) | 1.4 |  | 1.4(0.7) | 3.3 |  | 0.3(0.3) | 1.9 |  | 0.7(0.8) | 2.6 |  | 0.5(0.7) | 3.0 |
| [^18^F]THK5351 |  |  |  |  |  |  |  |  |  |  |  |  |  |  |  |  |  |
| Negative | 0.9(0.3) | 1.3 |  | 1.4(0.5) | 2.7 |  | 1.3(0.9) | 2.3 |  | 0.6(0.5) | 1.6 |  | 0.4(0.5) | 1.6 |  | 0.2(0.2) | 0.8 |
| Positive | 1.0(0.3) | 1.4 |  | 0.9(0.4) | 2.0 |  | 1.3(0.5) | 2.7 |  | 0.3(0.3) | 1.2 |  | 0.1(0.1) | 0.9 |  | 0.2(0.4) | 2.1 |

Supplementary Table 2. Registration errors for the corrected and uncorrected positron emission tomography images

|  | Translation [mm] | | | | | | | |  | Rotation [degree] | | | | | | | |
| --- | --- | --- | --- | --- | --- | --- | --- | --- | --- | --- | --- | --- | --- | --- | --- | --- | --- |
| default  SPM | x | |  | y | |  | z | |  | x | |  | y | |  | z | |
|  | Ave (SD) | Max |  | Ave (SD) | Max |  | Ave (SD) | Max |  | Ave (SD) | Max |  | Ave (SD) | Max |  | Ave (SD) | Max |
| [^11^C]PiB |  |  |  |  |  |  |  |  |  |  |  |  |  |  |  |  |  |
| Corrected | 0.2(0.3) | 1.3 |  | 0.5(0.5) | 2.5 |  | 0.9(1.6) | 2.6 |  | 0.8(0.6) | 2.4 |  | 0.3(0.3) | 1.2 |  | 0.2(0.2) | 0.9 |
| Uncorrected | 0.2(0.3) | 1.5 |  | 0.7(0.7) | 3.0 |  | 1.6(1.3) | 4.9 |  | 0.7(0.8) | 3.2 |  | 0.3(0.3) | 1.3 |  | 0.2(0.2) | 0.8 |
| [^18^F]florbetapir |  |  |  |  |  |  |  |  |  |  |  |  |  |  |  |  |  |
| Corrected | 0.1(0.1) | 0.3 |  | 0.2(0.1) | 0.4 |  | 0.8(0.6) | 1.6 |  | 0.2(0.2) | 0.9 |  | 0.1(0.1) | 0.3 |  | 0.1(0.1) | 0.3 |
| Uncorrected | 0.1(0.0) | 0.3 |  | 0.3(0.2) | 0.7 |  | 2.0(1.0) | 2.8 |  | 0.1(0.1) | 0.3 |  | 0.1(0.1) | 0.4 |  | 0.1(0.1) | 0.4 |
| [^11^C]PBB3 |  |  |  |  |  |  |  |  |  |  |  |  |  |  |  |  |  |
| Corrected | 0.7(0.6) | 2.3 |  | 1.6(0.8) | 3.0 |  | 1.7(1.3) | 4.2 |  | 0.9(0.8) | 3.4 |  | 1.1(1.1) | 3.7 |  | 1.0(0.8) | 2.9 |
| Uncorrected | 0.6(0.5) | 2.3 |  | 2.2(1.1) | 3.9 |  | 4.2(2.1) | 7.7 |  | 1.5(0.9) | 4.4 |  | 1.1(1.0) | 3.5 |  | 0.8(0.8) | 2.9 |
| [^18^F]THK5351 |  |  |  |  |  |  |  |  |  |  |  |  |  |  |  |  |  |
| Corrected | 0.2(0.3) | 1.3 |  | 0.5(0.3) | 1.2 |  | 0.9(0.9) | 3.1 |  | 1.1(0.7) | 3.1 |  | 0.4(0.4) | 1.8 |  | 0.4(0.4) | 1.9 |
| Uncorrected | 0.3(0.3) | 1.3 |  | 0.7(0.5) | 2.2 |  | 2.4(1.9) | 7.1 |  | 0.9(0.7) | 2.6 |  | 0.4(0.5) | 2.2 |  | 0.4(0.5) | 2.1 |
|  |  |  |  |  |  |  |  |  |  |  |  |  |  |  |  |  |  |
|  |  |  |  |  |  |  |  |  |  |  |  |  |  |  |  |  |  |
|  | Translation [mm] | | | | | | | |  | Rotation [degree] | | | | | | | |
| Modified  SPM | x | |  | y | |  | z | |  | x | |  | y | |  | z | |
|  | Ave (SD) | Max |  | Ave (SD) | Max |  | Ave (SD) | Max |  | Ave (SD) | Max |  | Ave (SD) | Max |  | Ave (SD) | Max |
| [^11^C]PiB |  |  |  |  |  |  |  |  |  |  |  |  |  |  |  |  |  |
| Corrected | 0.9(0.3) | 1.3 |  | 0.9(0.3) | 1.3 |  | 1.3(0.5) | 2.2 |  | 0.2(0.2) | 1.1 |  | 0.2(0.2) | 1.6 |  | 0.1(0.1) | 1.0 |
| Uncorrected | 0.9(0.4) | 1.4 |  | 0.6(0.3) | 1.3 |  | 0.8(0.7) | 3.9 |  | 0.5(0.5) | 2.0 |  | 0.2(0.3) | 1.6 |  | 0.2(0.3) | 1.6 |
| [^18^F]florbetapir |  |  |  |  |  |  |  |  |  |  |  |  |  |  |  |  |  |
| Corrected | 1.0(0.1) | 1.3 |  | 0.9(0.3) | 1.3 |  | 0.9(0.6) | 2.5 |  | 0.5(0.6) | 1.9 |  | 0.1(0.2) | 0.8 |  | 0.2(0.3) | 1.0 |
| Uncorrected | 0.9(0.1) | 1.3 |  | 0.6(0.4) | 1.3 |  | 0.9(0.6) | 2.5 |  | 0.3(0.4) | 1.6 |  | 0.2(0.3) | 1.6 |  | 0.3(0.3) | 1.2 |
| [^11^C]PBB3 |  |  |  |  |  |  |  |  |  |  |  |  |  |  |  |  |  |
| Corrected | 0.6(0.5) | 1.4 |  | 0.7(0.4) | 1.6 |  | 1.3(0.7) | 3.3 |  | 0.3(0.4) | 1.9 |  | 0.6(0.6) | 2.6 |  | 0.6(0.9) | 3.2 |
| Uncorrected | 0.6(0.5) | 1.8 |  | 0.9(0.5) | 2.1 |  | 1.5(0.9) | 4.8 |  | 0.3(0.4) | 2.2 |  | 0.4(0.5) | 2.4 |  | 0.7(0.9) | 4.0 |
| [^18^F]THK5351 |  |  |  |  |  |  |  |  |  |  |  |  |  |  |  |  |  |
| Corrected | 1.0(0.3) | 1.4 |  | 1.0(0.5) | 2.7 |  | 1.3(0.6) | 2.7 |  | 0.3(0.4) | 1.6 |  | 0.2(0.3) | 1.6 |  | 0.2(0.3) | 2.1 |
| Uncorrected | 1.0(0.3) | 2.0 |  | 0.7(0.4) | 1.7 |  | 1.0(0.9) | 4.7 |  | 0.5(0.5) | 1.9 |  | 0.3(0.4) | 1.8 |  | 0.3(0.4) | 1.9 |
